# Isolated Subcutaneous Mass of the Scalp as Initial Presentation of Metastatic Squamous Cell Carcinoma of the Lung

**Published:** 2013-11-08

**Authors:** Lauren V. Kuykendall, Jessica A. Ching, Wyatt G. Payne

**Affiliations:** ^a^Plastic Surgery Section, Bay Pines VA Healthcare System, Bay Pines; ^b^Division of Plastic Surgery, University of South Florida College of Medicine, Tampa, Fla

**Keywords:** cutaneous metastasis, lung cancer, scalp, squamous cell carcinoma, subcutaneous mass

## DESCRIPTION

A 66-year-old man presented with a symptomatic, mobile, 2.5-cm right forehead mass. Exploration for excision revealed a friable mass with invasion into the frontal bone. Magnetic resonance imaging revealed an intracranial mass. Positron emission tomographic scan indicated an additional lesion in the left lung. Lung mass pathology demonstrated identical immunohistochemical staining pattern to the frontal mass, suggesting a metastatic squamous cell carcinoma to the cranium with a primary pulmonary lesion.

## QUESTIONS

1. **What is the differential diagnosis of a subcutaneous mass and what is the common presentation of cutaneous metastases?**
2. **What is the incidence of cutaneous involvement as the initial presentation of metastatic disease?**
3. **When should preoperative imagining be considered?**
4. **What is the suggested treatment and prognosis of cutaneous metastasis?**

## DISCUSSION

A systematic approach to evaluating subcutaneous masses can help determine an appropriate differential diagnosis. One approach is to divide the possible diagnoses into categories such as mesenchymal tumors (lipoma, angiomas, liposarcoma, neurofibroma, etc), skin appendage lesions (epidermal inclusion cyst, pilomatricoma, cystadenoma, cylindroma, syringoma), metastatic tumors (carcinoma, melanoma, myeloma), other tumors or tumorlike lesions (myxoma, lymphoma, granuloma annulare), and inflammatory lesions (cellulitis, fasciitis, adenitis, abscess).^1^ The likelihood of these lesions as a diagnosis can be further arranged on the basis of the age of the patient, anatomic location, and clinical manifestations. In the case described earlier, based on age in the 60s, male gender, location on the forehead (or in the head and neck region), and relatively mobile, small, isolated mass, the most common diagnoses include lipoma, epidermoid inclusion cyst, carcinoma, and sarcoma. Clinically, cutaneous metastases often present as multiple, nontender, dome-shaped nodules that may be red, purple, or skin colored and can vary in size from 1 to 3 cm^2^. Often the lesions appear near the anatomical site of the primary tumor but can be found anywhere in the body.^2^^,^^3^ Approximately, 5% involve the scalp, and of those, lung and kidney in men and breast in women are the most prevalent primary lesions.^2^^,^^3^ Histologically, metastatic carcinoma is usually located in the mid-dermis or subcutaneous region and the epidermis is usually normal.^2^

The prevalence of cutaneous metastases of visceral tumors amounts to roughly 2% of all skin tumors.^4^ The incidence varies and ranges from 0.2% to 10%.^4^ A meta-analysis performed in 2003, which reviewed 20,380 cases, reported an overall incidence of cutaneous metastasis of 5.3%.^5^ A retrospective analysis of 100,453 cases by Saeed et al^3^ reported 28.6% of cutaneous metastasis emanated from the lungs, which was the most common primary lesion, with the head and neck as the cutaneous site of involvement in 28%. Saeed et al^3^ also reported cutaneous metastasis as the first indication of internal malignancy in 7.8% of the cases. Adenocarcinomas including large intestine, lung, and breast represent approximately 60% to 70% of cutaneous metastases.^2^ Squamous cell carcinomas including oral cavity, lung, and esophagus account for approximately 15% of metastatic disease in the skin, with the remainder of metastatic skin lesions composed of melanoma and other anaplastic tumors.^2^

In isolated cases, subcutaneous masses may represent a more complex diagnosis requiring further work-up before undergoing surgical excision. In general, small subcutaneous nodules do not require preoperative imagining. However, lesions that are firm, adherent to underlying tissue, or symptoms suggesting involvement of adjacent structures should be further evaluated with imaging prior to surgical intervention.^1^^,^^2^^,^^3^

Cutaneous metastases are a poor prognostic indicator, with an average survival time after diagnosis of approximately 3 to 6 months.^2^ According to Saeed et al, the average survival time varies slightly in patients with single (7.9 months) compared to multiple (5.5 months) sites of cutaneous metastasis.^2^ Treatment options almost exclusively pursue palliative goals including debulking of the tumor or radiation therapy in addition to the standard treatment for the primary malignancy.^4^ The patient in the case mentioned previously underwent a right frontal craniectomy for resection of the metastatic lesion with subsequent chemotherapy and radiation. Despite all treatment efforts, he expired 8 months following his initial diagnosis.

## Figures and Tables

**Figure 1:**
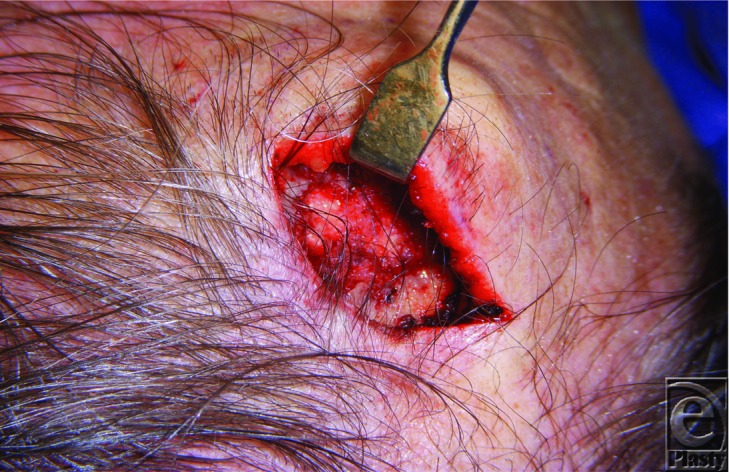
Intraoperative photograph demonstrating disruption of anterior table of frontal bone following debulking of subcutaneous mass.

**Figure 2:**
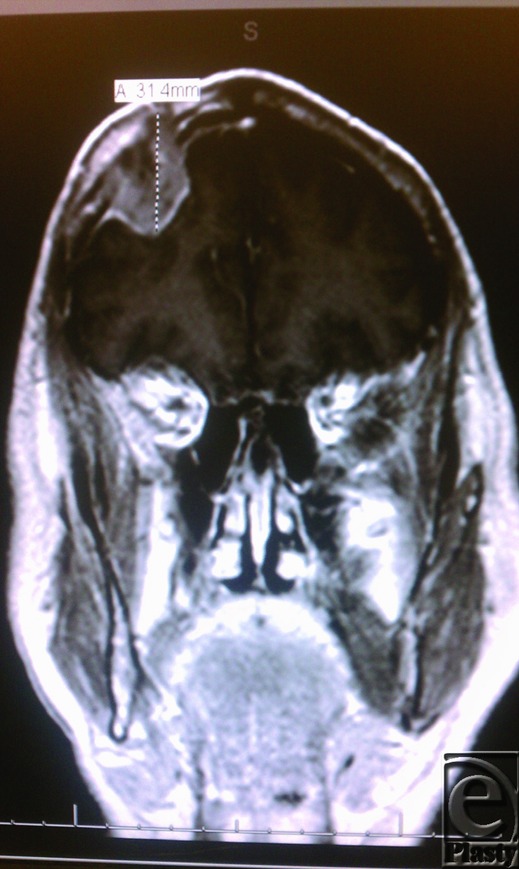
Left image: Magnetic resonance imaging coronal image of the head demonstrating enhancing mass of the right frontal bone with epidural extension into the extra-axial space overlying the right frontal lobe with mild associated mass effect.

**Figure 3:**
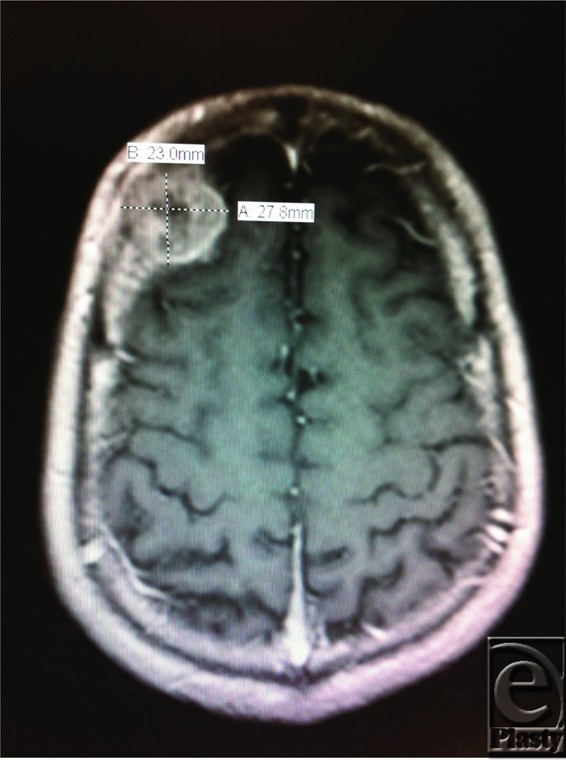
Right image: MRI of the head axial view showing 2.3 × 2.8 cm^2^ intracranial mass. Images taken following subcutaneous incisional biopsy.

**Figure 4:**
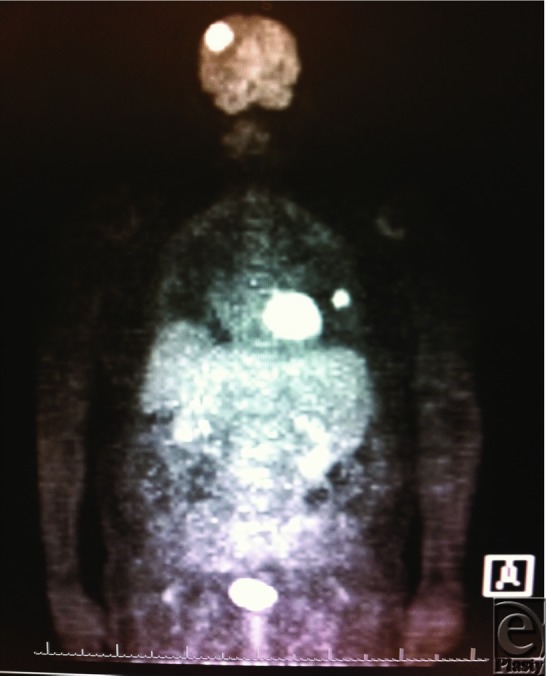
Positron emission tomographic scan demonstrating fluorodeoxyglucose avid left pulmonary nodule and right frontal mass.

## References

[1] Beaman FD, Kransdor MJ, Andrews TR, Murphey MD, Arcara LD, Keeling JH (2007). Superficial soft-tissue masses: analysis, diagnosis, and differential considerations. Radiographics.

[2] Garcia-Zuazaga J, Ke MS, Willen M (2009). Epidermoid cyst mimicry: report of seven cases and review of the literature. J Clin Aesthetic Dermatol.

[3] Saeed S, Keehn CA, Morgan MB (2004). Cutaneous metastasis: a clinical, pathological, and immunohistochemical appraisal. J Cutan Pathol.

[4] Nashan D, Muller ML, Braun-Falco M, Reichenberger S, Szeimies RM, Bruckner-Tuderman L (2009). Cutaneous metastases of visceral tumors: a review. J Cancer Res Clin Oncol.

[5] Krathen RA, Orengo IF, Rosen T (2003). Cutaneous Metastasis: a meta-analysis of data. South Med J.

